# TAM Receptor Inhibition–Implications for Cancer and the Immune System

**DOI:** 10.3390/cancers13061195

**Published:** 2021-03-10

**Authors:** Pia Aehnlich, Richard Morgan Powell, Marlies J. W. Peeters, Anne Rahbech, Per thor Straten

**Affiliations:** 1 National Center for Cancer Immune Therapy (CCIT-DK), Department of Oncology, Copenhagen University Hospital Herlev, 2730 Herlev, Denmark; marlies.peeters@regionh.dk (M.J.W.P.); anne.rahbech@regionh.dk (A.R.); 2 Department of Immunology and Microbiology, Faculty of Health and Medical Sciences, University of Copenhagen, 2200 Copenhagen, Denmark

**Keywords:** TAM receptors, Axl, MerTK, PD-1, small molecule inhibitors, cancer

## Abstract

**Simple Summary:**

TAM receptors are a family of receptor tyrosine kinases, comprising Tyro3, Axl and MerTK. Their primary role is in digestion of dying cells by macrophages without alarming the immune system. TAM receptors are also expressed by cancer cells in which signaling is oncogenic, and for this reason there is growing interest and research into TAM inhibition. This approach to cancer treatment may, however, come into conflict with beneficial and costimulatory TAM receptor signaling in T cells and natural killer (NK) cells. The aim of this review is to explore in detail the effects of TAM receptor inhibition on cancer cells and immune cells, and how the ramifications of this inhibition may affect cancer treatment in humans.

**Abstract:**

Tyro3, Axl and MerTK (TAM) receptors are receptor tyrosine kinases which play important roles in efferocytosis and in the balancing of immune responses and inflammation. TAM receptor activation is induced upon binding of the ligands protein S (Pros1) or growth arrest-specific protein 6 (Gas6) which act as bridging molecules for binding of phosphatidyl serine (PtdSer) exposed on apoptotic cell membranes. Upon clearance of apoptotic cell material, TAM receptor activation on innate cells suppresses proinflammatory functions, thereby ensuring the immunologically silent removal of apoptotic material in the absence of deleterious immune responses. However, in T cells, MerTK signaling is costimulatory and promotes activation and functional output of the cell. MerTK and Axl are also aberrantly expressed in a range of both hematological and solid tumor malignancies, including breast, lung, melanoma and acute myeloid leukemia, where they have a role in oncogenic signaling. Consequently, TAM receptors are being investigated as therapeutic targets using small molecule inhibitors and have already demonstrated efficacy in mouse tumor models. Thus, inhibition of TAM signaling in cancer cells could have therapeutic value but given the opposing roles of TAM signaling in innate cells and T cells, TAM inhibition could also jeopardize anticancer immune responses. This conflict is discussed in this review, describing the effects of TAM inhibition on cancer cells as well as immune cells, while also examining the intricate interplay of cancer and immune cells in the tumor microenvironment.

## 1. Introduction

The TAM receptors—Tyro3, Axl, MerTK—are a family of receptor tyrosine kinases that were discovered three decades ago, and are broadly expressed by a variety of cells and tissues in the body [1]. Ligands for the receptors are protein S (Pros1) and growth arrest-specific protein 6 (Gas6). Pros1 acts as ligand for Tyro3 and MerTK, whereas Gas6 is ligand for all three TAMs. Optimal signaling through the receptor is induced only when Pros1 and Gas6 act as bridging ligands for phosphatidyl serine (PtdSer), typically expressed on the outer membrane of apoptotic cells [2]. This leads to homodimerization and autophosphorylation of the TAM and downstream signaling; the outcome of which is highly cell-dependent but goes through pathways such as MEK/ERK, PI3K/AKT and JAK/STAT [3]. TAM receptors and ligands Pros1 and/or Gas6 are often co-expressed, and signaling is largely associated with efferocytosis, whereby TAM receptors expressed by macrophages, via Pros1/Gas6, bind PtdSer exposed by apoptotic cells or cell debris. The resultant signaling directs macrophages to an M2 phenotype, ensuring silent removal of dead cells and repair of tissues [4]. TAM receptors are also essential in regulation and control of inflammatory immune responses. Innate immune cells express pattern recognition receptors, e.g., toll-like receptors (TLR), which recognize microbial structures and upon ligand engagement induce an immune response [5]. To prevent a superfluous immune reaction, TAM signaling in dendritic cells (DC) functions as negative feedback to TLR signaling by induction of SOCS1/SOCS3 expression, thereby dampening the inflammatory response [6]. Axl signaling in murine natural killer (NK) cells limits the functional capacity of the cell [7], and activated T cells secrete Pros1, which works as an additional feedback mechanism to DCs and limits the immune response in a TAM-dependent manner [8]. As discussed in more detail later, we have shown that activated T cells express MerTK, which upon activation delivers a costimulatory signal to the T cell [9].

**Figure 1:**
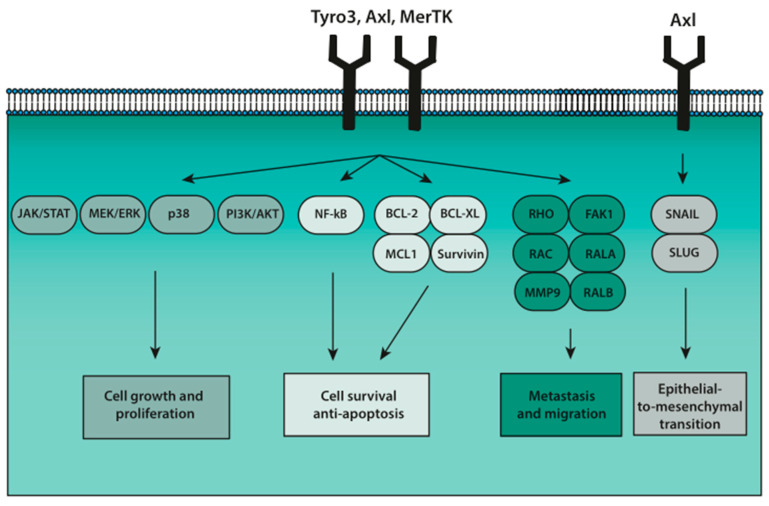
Activation of Tyro3, Axl and MerTK (TAM) receptors on cancer cells results in downstream oncogenic signaling. First, JAK/STAT, MEK/ERK, p38 and PI3K/AKT promote cell growth and proliferation in tumors. Second, cell survival and anti-apoptosis is induced through NF-κβ and BCL-2 pathways. Third, RHO and FAK1 signaling promote metastasis and migration. Lastly, epithelial-to-mesenchymal transition (EMT) is promoted through the SNAIL/SLUG signaling pathway.

MerTK and Axl are expressed in a wide range of cancer types, including non-small cell lung cancer [10], breast cancer [11], colorectal cancer [12], ovarian cancer [13] and melanoma [14], with 50–70% of patient samples being positive. Not only solid cancers, but also leukemias express Axl and MerTK [15,16]. For a comprehensive list of MerTK and Axl expression in cancers, refer to Graham et al., 2014 [4]. Cancer cells express both TAM molecules and their ligands, and, despite not being apoptotic, they also expose PtdSer on the outer membrane. This phenomenon of apoptotic mimicry was first described in virally infected cells as a means to dampen antiviral immune responses in the host [17]. PtdSer expression of cancer cells similarly dampens immune responses by interacting with TAM on antigen-presenting cells [18]. Furthermore, the co-expression of TAM, ligand(s) and PtdSer on cancer cells allows for autocrine signaling through the receptors, i.e., typical oncogenic signaling pathways like MEK/ERK, PI3K/AKT and JAK/STAT. In cancer cells, additional oncogenic pathways are associated with TAM receptor signaling. Anti-apoptosis and survival are promoted through activation of the NF-_K_B pathway and signaling via BCL-2 and ACK1 [10,19,20]. In regards to metastasis, TAM signaling regulates migration and invasion through promotion of proteins such as RHO and FAK1 [21] as well as MMP9 [22]. Axl additionally upregulates Snail and Slug, which induces epithelial-to-mesenchymal transition (EMT) [23] (see Figure 1).

Expression of TAM is associated with a worse disease outcome in a range of cancer diagnoses, both solid and hematological (reviewed in [4]), which has prompted the development of strategies to block or inhibit TAM signaling in cancer cells [24]. While most aspects of Tyro3 are understudied, data are accumulating concerning the complex biology of MerTK and Axl in cells, tissues, the immune system and cancer. In this review, we highlight the key roles of MerTK and Axl as both oncogenes and immune system regulators. We discuss the current standing and future prospects of MerTK and Axl inhibition in the treatment of cancer and the balancing act that must be maintained to limit any potential detrimental effects on immune cells.

## 2. MerTK

### 2.1. Targeting MerTK in Cancer

A wide range of cancers have been demonstrated to express high levels of MerTK, in both blood malignancies and solid cancers [24], resulting in gathering interest in the targeting of MerTK for cancer treatment. MerTK was first identified on a B lymphoblast library [25], and later characterization of acute myeloid leukemia (AML) and acute lymphocytic leukemia (ALL) showed expression in more than 80% of patient samples [15]. Importantly, numerous studies in various cancer diseases have been able to show that expression of MerTK is associated with a worse outcome [24]. Cancer cells that express high levels of TAM receptors, including MerTK, also express the ligands Gas6 and/or Pros1, as well as PtdSer on the outer membrane [26]. As a consequence, cancer cells express all the prerequisites to MerTK autosignaling, which has been demonstrated to assist in formation of metastases, chemoresistance and proliferation [4].

These findings have led to the development of MerTK-targeting therapies in the form of small molecule inhibitors as well as blocking antibodies. Preclinical studies have shown that MerTK inhibitors can be effective in certain MerTK-sensitive tumors. MerTK/FLT3-specific small molecule inhibitors UNC2025, MRX2843 and UNC1666 were shown to have significant effect on tumor growth of AML, ALL and melanoma in cell lines, murine models and primary patient tumor samples [27,28,29,30].

Apart from cancer-intrinsic features, MerTK signaling on tumor cells has been shown to also increase expression of PD-L1 [31], potentially limiting cytotoxic T cell antitumoral responses. Obviously, this phenomenon is associated with therapeutic implications, since the main breakthrough in cancer immunotherapy is treatment based on breach of the PD-L1/PD-1 axis between cancer cells and cytotoxic T cells. In addition, it has been demonstrated that MerTK signaling in APC promotes tolerogenic signaling and thus limits the ability of the APC to activate T cells [32]. Due to apoptotic mimicry, cancer cells seduce macrophages to silent phagocytosis via MerTK signaling instead of triggering an inflammatory response. Therefore, it is postulated that MerTK inhibition can be used to both control tumor growth directly and convert an immunosuppressive tumor microenvironment into an antitumoral microenvironment more prone to response to immunotherapy. To this end, Cook et al. demonstrated that MerTK knockout in a murine breast cancer model both reduced tumor growth and increased CD8 T cell infiltration [33]. Similarly, Tormoen et al. revealed that MerTK knockout in colorectal cancer and pancreatic ductal adenocarcinoma was responsible for control of tumor growth mediated by CD8 T cells and macrophages [34]. However, independent of any tumor model, Shao et al. showed that B cells in MerTK knockout mouse maintained normal activation, proliferation and antigen processing; however, their ability to activate T cells was decreased. The authors suggest this could be due to a reduced ability to effectively present antigen to CD4 T cells, illustrating that research on nontumoral effects of MerTK inhibition is required [35].

In recent years, the antitumor efficacy of small molecule inhibitors and MerTK-specific monoclonal antibodies (mAb) was demonstrated in a range of tumor models. In contrast to the previously mentioned studies that utilized MerTK knockout mouse models [33,34,35], MerTK inhibition alone was not always sufficient to impact on tumor outgrowth. It is, thus, highly important to point out that all the following studies required combination therapy involving αPD-1 treatment to facilitate antitumoral effects. Yokoyama et al. and Zhou et al. both studied PD-1 blockade and MerTK inhibition in the colorectal cancer mouse model, MC38. They demonstrated improved antitumor response, with increased Ki67+ CD8+ T cell infiltration and inflammatory cytokine profiles, when PD-1 blockade was used in conjunction with the MerTK small molecule inhibitor RDX106 or a MerTK monoclonal antibody [36,37]. In a lung xenograft carcinoma model, Caetano et al. showed that treatment involving αMerTK mAb and αPD-1 increased tumor infiltration of CD8 and NK cells. CD8+ CD103+ T cells were pointed out as a key phenotype that conveyed antitumoral effect [38]. A pan-TAM inhibitor, BMS 777607, was used by Kasikara et al. in a triple negative breast cancer model and it was revealed that this combination with PD-1 blockade again increased CD8 T cell infiltration, inflammatory cytokine profiles and, in this case, increased infiltration of Th1 type CD4 T cells [39]. The necessity of a combination therapy with anti-PD-1 is potentially due to the fact that PD-1 expression was increased on CD8+ T cells [36,37] and in the case of Kasikara et al., PD-L1 and PD-1 expression was increased in the tumor environment. It can therefore be posited that PD-1 blockade was required to unleash antitumor T cell response in these studies [39].

MerTK inhibition in murine tumor models has also revealed how macrophages and myeloid-derived suppressor cells (MDSCs) are affected in the context of an immune-driven antitumor response. A key mechanism is the ability of MerTK inhibition to switch M2 to M1 type macrophages and their relevant proinflammatory cytokine milieu [40]. Using MerTK inhibitor MRX2843, Lee-Sherrick et al. and Su et al. demonstrated in leukemia and glioblastoma models, respectively, that MerTK inhibition played a role in altering macrophage phenotype [41,42]. A switch from CD206+ M2-like macrophages to M1-like macrophages was reported with reduced PD-L1 expression, in conjunction with increased inflammatory cytokines and reduced regulatory T cell numbers. Holtzhausen et al. revealed that tumor-associated MDSCs express TAM receptors and that TAM inhibitor UNC4241, in conjunction with αPD-1, reduced tumor growth in melanoma models [43]. The MDSC’s suppressive capacity was functionally reduced and infiltration of CD8 T cells was increased. Du et al. utilized a broad spectrum TAM inhibitor to demonstrate that in lung and breast cancer models, MDSCs and M2-like macrophages were reduced along with reduced anti-inflammatory Arg-1 and IL-4 in the tumor-microenvironment [44]. However, they did show that PD-L1 and PD-1 were increased and therefore combination therapy with αPD-1 was required to sufficiently reduce tumor growth.

### 2.2. The Function of MerTK on T Cells

The wealth of evidence from combination therapy involving MerTK inhibition in murine models suggests this route of treatment is beneficial for CD8 T cell-mediated antitumoral responses. This is somewhat controversial as recent papers have demonstrated that MERTK is expressed by activated T cells [45]. Initially, MerTK expression on mouse splenocytes or human T cells was found to be absent following two-day stimulation with PMA/PHA [46,47]. However, Cabezón et al. clarified that human CD4+ T cells expressed MerTK three days after activation when using αCD3/CD28 [32]. This was supported by a paper from Peeters et al., who moreover exposed MerTK expression on human CD8+ T cells to have a late costimulatory role [9]. Inhibition of MerTK signaling by the inhibitor UNC2025 or by siRNA resulted in decreased release of interferon (IFN)-γ and had a negative impact on proliferation of human CD8+ T cells. This costimulatory role was contrary to its previously described role as a negative regulator of the innate immune response [6] and APC activation [32]. Thus, MerTK signaling has shown ambivalent roles depending on cell subset, species origin and type of T cell stimulation, which further complicates the idea of therapeutic targeting.

As previously mentioned, an interplay between MerTK and PD-1 has been suggested, mainly based on murine studies using combination therapies with αPD-1 [36,37,38,39]. This was further confirmed by unpublished data from our group. Flow cytometric staining of activated human T cells indicated co-expression of MerTK and PD-1, as MerTK positive cells showed significantly higher levels of PD-1 expression compared to MerTK negative cells. Additionally, the expression kinetics of both molecules seem to go hand in hand over several days. PD-1 is known to be expressed by activated T cells and to be dependent on TCR signaling. PD-1 is furthermore used as a marker of tumor-reactive T cells among tumor-infiltrating lymphocytes (TIL) [48]. Based on our previously described costimulatory role of MerTK and this well-known role of PD-1, the MerTK and PD1+ double positive T cells seem to have a tumor-reactive phenotype. If PD-1 is decreased upon MerTK inhibition, as described by Lee-Sherick et al. [41], it might thus result in less activated, less tumor-reactive T cells. While this might not pose a problem in short-term assays, as we have seen in most mouse model studies, it should perhaps be considered in the more long-term clinical setting.

MerTK signaling seems to have a regulatory role in T cell memory responses. Observations from Peeters et al. showed a significantly higher level of MerTK expression on the T_CM_ CD8+ subset [9]. This was further supported by data indicating that expression kinetics of MerTK follow the fraction of the central memory T cell (T_CM_) subset. Moreover, upon restimulation of the T cells, the percentage of MerTK-expressing cells increased significantly. Additional analysis of the T_CM_ subset upon restimulation revealed a higher fraction of T_CM_ within the MerTK-expressing cells (unpublished data). It, therefore, seems that MerTK signaling supports the formation of a central memory response. Thus, generation of long-term memory T cell responses could be jeopardized by MerTK inhibition. Importantly, it remains understudied if mouse T cells express MerTK upon activation. Surely, if this is not the case, this important role of MerTK in human T cells would not be illuminated in mouse tumor models.

### 2.3. MerTK in the Crossfire of Cancer Cells and T Cells

Due to the direct effect MerTK inhibitors have on their respective tumor targets, research into potential off-target effects on other cell types is scarce so far. Evidence suggests higher concentrations of inhibitors, i.e., >500 nM, impact colony formation of cord blood cells [27,28,29]. Since we know that T cells express MerTK and that it plays a role in costimulation and memory formation, further research needs to be undertaken to assess direct effects of new and potent MerTK-specific inhibitors. Two studies have briefly assessed the effect on CD8 T cells, suggesting no direct effect on CD8 T cell proliferation when their respective inhibitors were used [36,43]. However, the concentration tested was not stated or very low. Concentration is key in this respect, given that a balance needs to be struck between a direct tumor effect versus any detrimental effect on MerTK+ T cells. Since both cancer cells and immune cells express MerTK, it is important to investigate the role of MerTK signaling in the tumor context. It is firmly established that MERTK inhibition on cancer cells inhibits survival signaling pathways. Moreover, MerTK inhibition on APCs would block efferocytosis and render the cells more capable in priming T cell responses. On the other hand, blocking MERTK signaling on T cells would render the T cell less capable of cytokine production, proliferation and killing.

To answer this question of how MerTK inhibition may affect MerTK+ T cells in an antitumor cell response, we studied MerTK signaling in melanoma TILs by examining the impact of Pros1 [9]. Melanoma TILs are used for adoptive cell therapy (ACT) at our research center and TIL expansion represents a crucial step in the process since failure to grow cells obviously jeopardizes patient treatment. Clearance of Pros1 by an αPros1 antibody during TIL expansion resulted in a significant decrease in fold expansion rate. Similarly, we studied if addition of increasing Pros1 concentrations would impact the cytotoxicity of expanded TILs against an autologous melanoma cell line. These data indicated a trend, albeit non-significant, that higher Pros1 levels corresponded with enhanced killing activity of the T cells.

MerTK activation initiates a signaling cascade in cancer cells that classically promotes cancer cell survival, and in T cells provides a costimulatory signal. In the TME, this sets the stage for competition for Pros1 between cancer cells and T cells, and this balance may affect aspects of immune microenvironment resolution. In co-culture studies with the breast cancer cell line MDA-MB.231, which expresses high levels of MerTK, we could show that low concentrations of Pros1 resulted in an enhanced inhibition of T cell activation by the cancer cells. This competitive effect was abrogated when an excess amount of Pros1 was present in the co-culture media [9]. Cancer cell lines express a higher level of TAM receptors than activated T cells, which explains a higher Pros1 consumption by MDA-MB.231 cells compared to activated T cells. This shows how TAM expression by cancer cells, or any cell of the TME, could set the stage for ligand competition and Pros1 starving of T cells. One strategy to overcome this ligand competition could be to equip T cells with a constitutively active MerTK construct [49], thereby increasing MerTK signaling and rendering the T cell insensitive to Pros1 starvation.

## 3. Axl

### 3.1. Axl Drives Cancer Cell Survival, EMT and Chemoresistance

In recent years, Axl has been investigated to a great extent for its role as an oncogene and driver of cancer cell survival. It was this very role that led to the discovery of Axl in the first place; the gene was isolated from two patients with chronic myelogenous leukemia (CML) and shown to cause neoplastic transformation in NIH3T3 cells [50]. Following this discovery, a myriad of articles have been published to unravel Axl’s involvement in cancer cell survival and metastasis in many cancer types (as reviewed in [51]). Downstream signaling of Axl resembles many other receptor tyrosine kinase signaling pathways and their specific activation seems to be dependent on cell and tissue type [51]. In general, Axl mediates both tumor-proliferating and anti-apoptotic functions, which synergize in promoting cancer cell survival. In addition, Axl signaling has more specialized features in cancer cells, two of which will be highlighted in the following.

First, Axl has repeatedly been shown to be involved in epithelial-to-mesenchymal transition (EMT), thus being a crucial player in enabling cancer cell metastasis [52]. Axl has been pointed out both as an upstream regulator [23] and a downstream effector [53] of EMT. Asiedu et al. showed that vector-based overexpression of Axl in human breast epithelial cells resulted in downregulation of the epithelial marker E-cadherin and a concurrent upregulation of mesenchymal markers N-cadherin, Snail and Slug [23]. This EMT phenotype was suppressed when silencing Axl with shRNA or when blocking the NFκB pathway with proteasome inhibitor MG132. This elegantly demonstrated the role of Axl in inducing EMT, in part at least via activating NFκB. A recent study supported these results, as the ectopic expression of Axl in human gastric cancer cell lines also downregulated E-cadherin and upregulated the mesenchymal marker vimentin [54]. In both studies [23,54], inactivation of Axl in in vivo models slowed tumor outgrowth. As Axl is not only implicated as an inducer but also an effector of EMT, this strongly points towards a positive feedback loop to sustain the EMT phenotype of tumor cells. The induction of Axl by the EMT program has been observed in different tumor types, such as breast cancer [53,55], skin cancer [56], esophageal cancer [57] and ovarian cancer [58]. Axl signaling was shown to facilitate cancer cell invasion in esophageal cancer by increasing extracellular acidification by upregulation of lactate transporters. The increased extracellular pH, in turn, led to a peripheral distribution of lysosomes and enhanced lysosomal exocytosis and secretion of cathepsin B, a protease involved in extracellular matrix degradation. This paved the way for cancer cell migration from the primary site [57].

Second, expression of Axl plays a role in mediating chemoresistance, which is a major challenge in providing long-lasting treatment to cancer patients. Some cancers are more prone to chemoresistance than others and increased frequency of chemoresistance often goes hand in hand with a poorer prognosis. This, for instance, is the case for ovarian cancer, in which resistance to the first-line platinum-based chemotherapeutics is very common [13,59]. The chemoresistance against platins in ovarian cancer has been shown to be mediated by Axl by different mechanisms. Axl signaling was found to hamper intracellular accumulation of carboplatin in ovarian cancer cell line POV71-hTERT, thus keeping intracellular carboplatin concentrations at suboptimal levels [13]. Axl blockade in POV71-hTERT cells revealed an upregulation of multidrug resistance gene P-glycoprotein expression. P-glycoprotein is an ATP-dependent efflux pump, which could potentially export carboplatin from the cell. Therefore, this could indicate a pretarget resistance mechanism, as per classification of platinum resistance mechanisms by Galluzzi et al. [60]. Additionally, Axl has been implied in an off-target resistance mechanism against cisplatin: Axl seems to increase aerobic glycolysis of ovarian cancer cells, also known as the Warburg effect [59]. Specifically, Axl was shown to phosphorylate the pyruvate kinase isoform PKM2 at tyrosine 105, which favors ATP production by aerobic glycolysis over the Krebs cycle. This altered metabolism, with its increased energy production and biosynthesis, was suggested to compensate for the lethal cisplatin signaling. The diversity of chemoresistance mechanisms induced by Axl illustrates the key role that Axl plays in platinum resistance. Apart from ovarian cancer and platinum, Axl mediates chemoresistance in a range of other cancers and drugs. Just to name a few, Axl was shown to cause resistance of astrocytoma cell line A172 to carboplatin [61], resistance of FLT3-internal tandem duplication AML to quizartinib [62] or resistance of EGFR-mutated lung cancer to osimertinib [63].

Taken together, this shows that Axl signaling has a variety of beneficial effects on cancer cells that could be exploited therapeutically.

### 3.2. Targeting Axl on Cancer Cells and Its Implications

The strong oncogenic properties of Axl expression and signaling have led to the development of Axl inhibitors; one of the best known to date is bemcentinib (formerly BGB324 or R428). It has been shown to block proliferation and growth signaling [16] as well as tumor metastasis [52]. Furthermore, Axl inhibition enhances apoptosis mediated by chemotherapeutic drugs [64]. Due to the success of Axl inhibitors in preclinical models, some treatment prospects have been advanced into clinical testing. The lead candidate is bemcentinib, being tested in as much as ten clinical phase I/II studies registered on ClinicalTrials.gov (accessed on 28/10/2020). The clinical trials cover a wide range of tumor diagnoses, from brain cancer over triple-negative breast cancer to acute myeloid leukemia. Bemcentinib is being tested as a single-treatment agent as well as in combination with, e.g., chemotherapy, targeted therapy and immune checkpoint blockade. To date, only one study is registered as completed. TP-0903 is another candidate in clinical testing, comprising four registered in-patient trials. To our knowledge, only preliminary clinical data has been made available for these trials, showing some promising responses.

Besides merely being a treatment target, Axl has often been implied as a prognostic factor for cancer patients. High Axl expression is usually associated with a worse outcome [65]. These associations are mostly based on cancer gene signatures, allowing for determination of expression of Axl RNA. Axl RNA expression, however, has been shown to not directly correlate to Axl protein expression [66], which is necessary for functionality of the receptor. In their murine studies, Zagórska and colleagues demonstrated that Axl mRNA copy number is very similar in DCs and bone marrow-derived macrophages. Despite this, Axl protein expression is high on DCs, but low on bone marrow-derived macrophages, suggesting that post-transcriptional or post-translational mechanisms regulate Axl expression. Therefore, it should be considered that PCR-based evidence for high Axl expression on cancer cells does not necessarily translate into high expression of Axl protein.

Nevertheless, whether Axl is expressed on cancer cells or not, emerging evidence suggests that Axl inhibitors could affect cancer cells regardless of Axl expression. In a study by Chen et al., Axl inhibitor bemcentinib induced cancer cell apoptosis in cell lines, in which Axl was knocked down by a siRNA approach. They demonstrated that bemcentinib blocks Axl phosphorylation short-term and induced Axl upregulation, while still causing increased apoptosis—this increased apoptosis was observed even in Axl knockdown cell lines [67]. This was traced down to protonation of bemcentinib following accumulation in the lysosomes, which altered lysosomal pH. In turn, the altered pH disturbed lysosomal degradation leading to vacuolization, and eventually triggered cell death. This is underlined by R428′s molecular structure, which resembles other lysomotropic agents that induce vacuolization. An important consideration is that the entrapment of bemcentinib in lysosomes could explain the merely transient effect as an Axl inhibitor. Furthermore, Chen et al. showed that bemcentinib initiated autophagy, possibly as a feedback signal to compensate for the disrupted autophagic protein processing in the lysosomes, which, however, aggravates the problem. Increased autophagic flux was also observed upon treatment of renal cancer cells with bemcentinib [64]. These results, though, stand in contrast to a study by Lotsberg et al. [68]*,* which showed that bemcentinib abrogates autophagy in non-small cell lung cancer (NSCLC) and thus induces immunogenic cell death. These differences could be related to different cancer cell lines studied, but also illustrate very well that the mechanism(s) of small molecule Axl inhibitors are not entirely clarified yet.

Upregulation of Axl caused by a small molecule Axl inhibitor as described by Chen et al. was later confirmed in another study with Axl TKI BMS777607 [69]. The authors found that Axl phosphorylation upon Gas6 stimulation is necessary for the protein to be ubiquitinylated, internalized and degraded by the lysosomal pathway. However, blocking receptor activation with a tyrosine kinase inhibitor also blocks this required phosphorylation, which leads to an accumulation of an inactive Axl receptor on the cell surface. Phosphorylation of this receptor can still take place, even after days, which causes severe consequences in the tumor microenvironment. In sections of tumors where the drug gradient might fall below inhibitory concentrations, accumulated Axl receptors may start to signal again, reversing any desired effect of treatment.

### 3.3. Axl Plays a Role in Immune Homeostasis

Axl can be expressed by various immune cells, which implies that targeting of Axl in cancer therapy may also have an impact on the immune system.

In DCs, Axl signaling plays a major role in terminating immune responses at the end of an infection [6]. TLR activation on DCs upregulates Axl expression via STAT1 signaling. Upon activation, Axl hijacks the IFNAR-STAT1 signaling cassette to induce SOCS1 and SOCS3. These molecules then suppress TLR signaling, terminating DC priming of T cells. Blocking this pathway could avoid the negative feedback mechanism, allowing continued immune responses.

Macrophages are another cell type in the myeloid compartment that expresses Axl. TAM receptor expression on macrophages is crucial in the process of efferocytosis, the immune-quiescent phagocytosis of apoptotic cells. While MerTK surely takes the lead role in this process [70], nuclear receptor signaling following efferocytosis is suggested to also upregulate Axl transcription in macrophages [71]. Blockade of Axl, or TAM receptors in general, can thus hinder efferocytosis [72]. Consequently, phagocytosis of (dying) tumor cells would trigger an inflammatory response, which could attract more immune cells to the crime scene. In the tumor context, Axl is also suggested to play a role in polarization of tumor-associated macrophages (as reviewed in [73]). There is conflicting evidence on Axl’s involvement; while Axl inhibition with R428 decreased M2-associated factors (IL-10 and TGF-β) in mineral trioxide aggregate-treated THP-1 cells [74], blocking Axl with a monoclonal antibody in an MDA-MB231 xenograft model decreased M1-associated factors (IL-6, TNF-α, G-CSF) [75].

Furthermore, Axl signaling has been implied in optimal NK cell differentiation [76,77]. It should be considered that Axl inhibition could therefore hamper differentiation from immature NK cells into cytotoxic NK cells. Similar to DCs, Axl could possibly also be implicated in the negative regulation of NK cells [78]. Upon stimulation with interleukin-15, NK cells were shown to have reduced IFN-γ secretion if pretreated with type I interferons. This went hand in hand with an upregulation of Axl on NK cells, leading to the suggestion that Axl might be the mediator of diminished IFN-γ secretion. However, this mechanism remains to be fully investigated.

### 3.4. Axl Inhibition and the Effect on the Immune Response against Cancer

The previous sections have illustrated that Axl plays a role both in tumor biology and in the immune system. In an era during which the immune system’s ability to control tumor growth is increasingly harnessed by immunotherapies, it is worth investigating which role Axl plays in this interaction between cancer and immune cells.

Besides its direct effects on cancer cell survival and invasion, blocking of Axl signaling has other antitumor effects, which render the tumor more susceptible to immune cell killing. Recently, Axl targeting was discovered to have a positive impact on the expression of ICAM-1 and ULBP-1 on lung carcinoma [79]. ICAM-1 is an adhesion molecule implicated in lymphocyte tissue migration and stabilization of cell–cell junctions, whereas ULBP-1 is a ligand for NKG2D, an activating NK receptor. By upregulation of these molecules, the lung carcinoma cell line clones displayed increased sensitivity to NK- and T cell-mediated killing. Likewise, Woo et al. found that R428 could enhance the effect of TRAIL-mediated killing [64]. TRAIL is a death receptor ligand, which is expressed by various immune cells. In their study, apoptosis induced by recombinant TRAIL was increased by R428 in a variety of cancer cell lines. They demonstrated that R428 induced upregulation of miR-708, which inhibits c-FLIP expression. Additionally, R428 triggered proteasomal degradation of survivin. Both c-FLIP and survivin are anti-apoptotic proteins, which explains why their downregulation allows increased apoptosis.

Apart from targeting tumor cells, Axl inhibition also has direct effects on the immune system, which could aid the immune cells in destructing the cancer cells. Specifically, the role of Axl in terminating immune responses should be discussed. Both in DCs and in NK cells, Axl has been implied in negative feedback loops and breaking these loops could restore essential immune responses in the tumor microenvironment. This concept is emphasized by a study by Guo et al., in which they could show that Axl inhibition (both by bemcentenib and SGI-7079) reprogrammed the tumor microenvironment in two different mouse tumor models [80]. They found that Axl inhibition caused a reduction of monocytes/macrophages and granulocytes in the tumor, but an accumulation of CD103+cDCs. These cDCs also expressed higher amounts of activation markers CD40 and CD86. Concomitantly, this led to increased infiltration of activated proliferating T cells (Ki67 and CD69 positive) to the tumor. This could suggest that the activated DCs induced a heightened T cell response, which was also underlined by higher Th1-associated gene expression in the tumor. However, this adaptive immune response was counteracted by PD-L1 upregulation on the tumor cells. This went hand in hand with an induction of PD-1 expression on the tumor-infiltrating T cells, which suggested a formation of an adaptive immune resistance. PD-1 blockade could reverse this acquired resistance, to the degree that a third of mice treated with αPD-1 mAb and an Axl inhibitor were cured and resistant to rechallenge.

Similarly, in mice, TAM receptor signaling was suggested to attenuate NK cell responses via ubiquitin ligase Cbl-b. Hence, inhibition of TAM receptors, including Axl, could enhance NK cell function, shown by a decreased metastatic load in mice models [7,81]. Moreover, blockade of Axl ligand Gas6 signaling was also shown to activate NK cells, which further underlined this mechanism in murine tumors [82]. However, expression of Axl on mature human NK cells is debated: mRNA expression of Axl was shown to disappear on day 10 of NK cell differentiation from CD34+ human pluripotent stem cells [76]. On the contrary, a study on NK cells isolated from peripheral blood mononuclear cells found that a fraction of NK cells expressed Axl (approx. 1%) and that this expression was tripled by IFN-β stimulation [78]. Still, this raises questions about the functional relevance of Axl on human NK cells, and thus the effect of Axl inhibitors.

## 4. Conclusions

**Figure 2:**
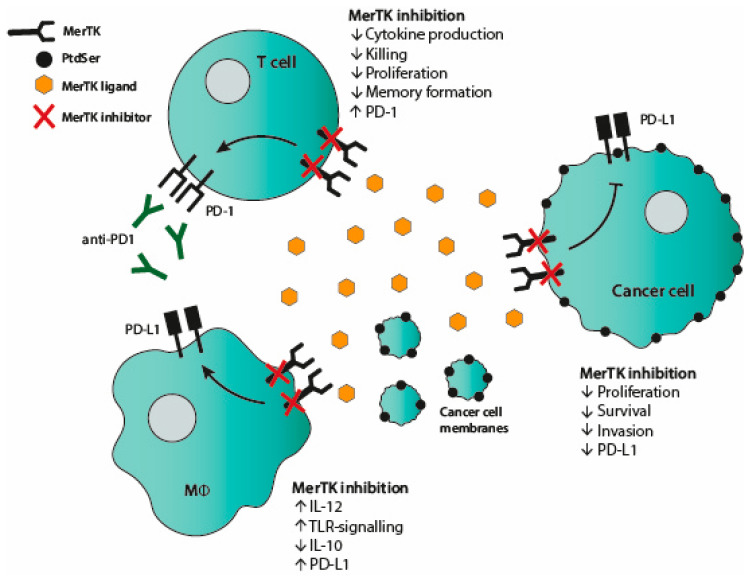
MerTK inhibition affects cancer cells, macrophages and T cells. In cancer cells, inhibition of MerTK signaling leads to reduced proliferation, survival and invasion. It also hampers upregulation of PD-L1 in cancer cells. In macrophages, MerTK inhibition increases IL-12 production, toll-like receptors (TLR) signaling and PD-L1 expression, while decreasing IL-10 production. MerTK blockade in T cells results in reduced cytokine production, cytotoxicity, proliferation and memory formation. It also induces expression of PD-1, thus, anti-PD-1 treatment should be considered in combination with MerTK inhibition.

**Figure 3:**
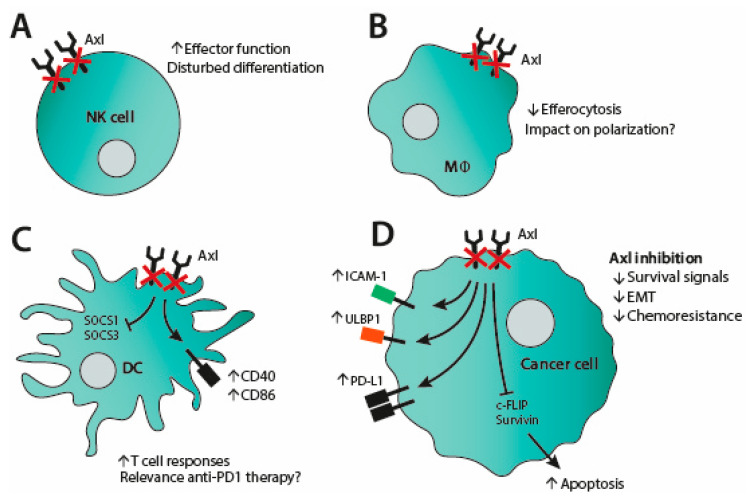
Axl inhibition influences both cancer and immune cells. (**A**) Blockade of Axl signaling in natural killer (NK) cells enhances effector function but has also been implied to disturb NK cell differentiation. (**B**) In macrophages, Axl inhibition leads to reduced efferocytosis and could also impact polarization. (**C**) Axl inhibition in dendritic cells (DCs) blocks SOCS1/3 signaling and upregulates CD40 and CD86. This results in increased T cell responses, which might need to be unleashed by PD-1 blockade. (**D**) Inhibition of Axl signaling in cancer cells diminishes survival signals, EMT as well as impedes chemoresistance. It also induces upregulation of ICAM-1, ULBP1 and PD-L1, while it downregulates c-FLIP and survivin and thus enhances apoptosis.

Thirty years after their characterization, TAM receptors are now recognized as important molecules in a range of normal physiological functions, e.g., efferocytosis, tissue repair, innate immune regulation and costimulation on T cells. TAM receptors also play important roles in cancer biology, which has prompted investigations on inhibition of mainly Axl and MerTK as a therapeutic strategy. To this end, various small molecule inhibitors have been developed, alongside a smaller number of blocking antibodies. In vitro inhibition of both MerTK and Axl has a negative impact on cancer cell survival, with enhanced apoptosis and diminished proliferation (see Figure 2 and Figure 3 for MerTK and Axl inhibition, respectively). Blocking of Axl also impedes EMT and reverses chemoresistance. The favorable attributes of TAM inhibition could also be translated in vivo, since blocking TAM receptors prevented tumor outgrowth in several murine studies. In these studies, increased inflammatory tumor cell killing was observed when Axl or MerTK signaling was abrogated by small molecule inhibitors, blocking antibodies or gene knockout. The heightened immune response is mainly ascribed to an increased CD8 T cell infiltration. MerTK inhibition is also associated with M1 macrophage polarization and a reduced number of MDSCs (see Figure 2). Axl inhibition, in contrast, is suggested to block negative feedback loops in DCs and NK cells (see Figure 3). Despite inducing an inflammatory tumor microenvironment, TAM inhibition leads to an adaptive resistance to immune cell killing by upregulating molecules of the PD-1/PD-L1 axis. Therefore, combining TAM inhibition with anti-PD-1 blockade seems necessary and has proven efficacious in mouse models. Still, the desired effect of TAM inhibition, even as combination therapy, may come into conflict with undesirable effects on the immune system: Axl signaling plays a role in NK cell differentiation and MerTK signaling provides costimulation to T cells. Indeed, our research has demonstrated direct adverse effects of MerTK inhibition on T cells, including reduced proliferation and cytokine production (see Figure 2). Furthermore, MerTK-positive T cells that are restimulated generate a significantly increased central memory pool. Upon use of MerTK inhibitors, this mechanism may be disrupted, favoring short-lived effector T cell responses over generation of memory pools. While this may result in effective treatment in the short term, an insufficient adaptive T cell memory pool may fail to prevent relapse in the long term. Further study is required to fully interrogate the duality between beneficial and detrimental effects of MerTK and Axl inhibition in cancer treatment and its consequences for the immune system.

Clinical trials are ongoing to investigate the therapeutic potential of Axl and MerTK small molecule inhibitors in cancer and data from these trials will provide valuable insights into the precise mechanism of action. Preclinical data have shown that Axl small molecule inhibitors likely affect cellular degradation pathways, even independent of Axl expression. This illustrates the need for careful monitoring of early clinical data to harness merely the beneficial effects of TAM small molecule inhibitors. In the future, this will possibly prepare the platform for intelligent combination treatments with conventional therapies, including immunotherapy.
